# Characterization of side population cells isolated from the gastric cancer cell line SGC-7901

**DOI:** 10.3892/ol.2013.1103

**Published:** 2013-01-02

**Authors:** RONG LI, XIAOLING WU, HUANG WEI, SHANGKUN TIAN

**Affiliations:** 1 Department of Gastroenterology, The Second Affiliated Hospital of Chongqing Medical University, Chongqing 400010;; 2 University Health Center, Southwest University of Political Science and Law, Chongqing 401120;; 3 Department of Gastroenterology, The First People’s Hospital of Jiulongpo of Chongqing, Chongqing 400050, P.R. China

**Keywords:** SGC-7901 cells, tumor stem cells, side population cells, Musashi-1, CD44, ABCG2, Bcl-2

## Abstract

Side population (SP) cells are a subset of stem cells that have been isolated from several different gastrointestinal cancer cell lines. Using flow cytometry and the DNA-binding dye Hoechst 33342, we isolated SP cells from SGC-7901 human gastric tumor cell lines and found that they comprise 2.3±0.78% of the tumor cells. Using the Cell Counting Kit-8 (CCK-8) assay, we demonstrated that SP cells have a stronger proliferative activity than non-SP cells. Additionally, we observed tumor mass formation following the cultivation of SP cells in serum-free medium, indicating the capability of these cells for self-renewal. SP cells were observed to undergo non-symmetrical division, which is characteristic of stem cells. A drug resistance assay revealed that SP cells have a high survival rate when exposed to the chemotherapy drug 5-fluorouracil; the results of western blot analysis suggest that this stems from the abundant expression of the chemoresistance-associated proteins ABCG2 and Bcl-2. We also used fluorescence quantitative PCR to reveal that SP cells have relatively high expression levels of the stem cell-related genes Musashi-1 and CD44. *In vivo* experiments in mice revealed that the subcutaneous injection of 2×10^3^ SP cells resulted in the formation of tumors, while the injection of 2×10^4^ non-SP cells did not. Cumulatively, our results suggest that gastric tumorigenesis associated with SGC-7901 may partly be driven by the activity of SP cells, which exhibit certain biological characteristics of stem cells. Our results also show that the SP cell sorting method is an effective means for isolating and identifying gastric cancer stem cells during early screening.

## Introduction

Side population (SP) cells are a small group of cells that stain faintly or not at all when treated with Hoechst 33342 dye. These cells are typically isolated by flow cytometry (FCM) using the method described by Goodell *et al* in a study of murine bone marrow (1). This technique has been used to sort SP cells from various types of cancer, including gliomas (2,3), and breast (4), colon (5,6), lung (7) and liver (8,9) cancer.

SP cells demonstrate self-renewal and multiplex differentiation potential. Further, xenograft experiments have revealed that these cells exhibit stem cell characteristics, including high *in vitro* proliferation ability and a strong tumor-forming ability. Although SP cells have been isolated and identified from several different cell lines of gastrointestinal cancer (10), there has been relatively little research conducted on SP cells in SGC-7901 cell lines from human gastric tumors.

The aim of the current study was to isolate and characterize SP cells from SGC-7901 cell lines. Specifically, we used the SP cell sorting method to isolate SP cells in order to investigate their proliferation, self-renewal, chemoresistance and differentiation properties. We hope that this information will lay the foundation for further gastric cancer stem cell research.

## Materials and methods

### 

#### Cells and experimental animals

The human gastric cancer cell strain SGC-7901 was donated by Dr Yan Xuedong from the First Affiliated Hospital of Chongqing Medical University. *In vivo* experiments were performed on 18 female specific pathogen-free (SPF) Balb/c nude mice (4–6 weeks old) that had been purchased from the Laboratory Animal Center of the Third Military Medical University (Chongqing, China). The breeding and use of the experimental animals were in accordance with the reviewed principles designated by the Ethics Committee of the Third Military Medical University.

#### Reagents

Trypsin-ethylenediaminetetraacetic acid (trypsin-EDTA), RPMI-1640 medium and fetal bovine serum (FBS) were purchased from HyClone Laboratories (Logan, UT, USA). Additionally, Hoechst 33342 and verapamil were purchased from Sigma (St. Louis, MO, USA), while epidermal growth factor (EGF) and basic fibroblast growth factor (b-FGF) were purchased from Peprotech (Rocky Hill, NJ, USA). Cell Counting Kit-8 (CCK-8), rabbit anti-human ABCG2 and rabbit anti-human Bcl-2 antibodies were purchased from Boster Biological Technology Ltd. (Fremont, CA, USA). Furthermore, TRIzol reagent was purchased from Invitrogen (Carlsbad, CA, USA), while the retrovirus kit and the Thunderbird SYBR qPCR mix were purchased from Toyobo (Osaka, Japan).

### Experimental methods

#### Cell cultures

SGC-7901 cells were cultured in RPMI-1640 medium with 10% FBS, 100 U/ml penicillin G and 100 *μ*g/ml streptomycin. In all experiments, the cells were cultured in a hatch box at 37°C, 5% CO_2_ and 95% humidity. Passage was completed in 3–4 days, and all experiments were performed on cells in the exponential growth phase.

#### Detection and sorting of SP cells by FCM

Cells were digested with 0.25% trypsin-EDTA and then centrifuged for 5 min at 1,000 rpm. The cells were subsequently suspended in phosphate-buffered saline (PBS) containing 5% FBS, then stained with Hoechst 33342 at a concentration of 10 *μ*g/ml, and incubated for 90 min at 37°C either alone or with 100 *μ*M verapamil. During the incubation, the cells were shaken every 10 min. A second round of centrifuging was performed for 5 min at 1,000 rpm, then the cells were suspended in PBS with 5% FBS at a concentration of 1×10^7^ cells/ml. The solution was poured through a 400-mesh screen filter and then stored in the dark at 4°C. Next, samples were dyed with 1 *μ*g/ml propidium iodide (PI) for 5 min to remove the dead cells. The remaining cells were sorted using a flow cytometer (FACS Aria II; BD Biosciences; Franklin Lakes, NJ, USA). The Hoechst 33342 dye was excited at 355 nm and its dual-wavelength fluorescence was analyzed (blue, 450 nm; red, 675 nm).

#### In vitro proliferation activity assay

After centrifuging and suspension, the acquired SP and non-SP cells were inoculated in a 96-well plate at 2×10^2^ cells/well, and then cultured in a CO_2_ incubator. Each group was set up in triplicate. Cell proliferation activity tests were performed every day for 7 days. CCK-8 solution (10 *μ*l) was added to each well and the plate was placed in a CO2 incubator for 3 h. The optical density (OD) was determined at 450 nm. These data were used to calculate cell growth curves based on the mean OD_450_ and standard deviation values for each well.

#### Observation of tumor mass formation ability in a serum-free medium

The SP and non-SP cells were each inoculated in 3 ml RPMI-1640 serum-free medium in non-adsorption petri dishes at a density of 200 cells/dish. Following treatment of the cultures with 20 ng/ml EGF and 10 ng/ml b-FGF, the plates were placed in a CO_2_ incubator. Tumor mass formation was observed under a microscope after 7 days.

#### Appraisal of non-symmetrical division ability

After centrifuging, the SP cells and non-SP cells were resuspended in RPMI-1640 medium containing 10% FBS for 1 week of routine culture. After this time, the SP sorting method was applied to the two groups to re-evaluate the proportion of SP cells present in the culture.

#### Cell resistance experiment

SP and non-SP cells were seeded in 96-well plates at a concentration of 1,000 cells/plate. Following 24 h of cultivation, 5-fluorouracil (5-FU) was added to all cultures to a final concentration of 10 *μ*g/ml. The plates were placed in a hatch box for 48 h. A solution of CCK-8 (10 *μ*l) was then added to each well, and the plates were incubated for an additional 3 h. The mean and standard deviation of absorbance at OD_450_ were then calculated. Cell resistance in both groups was calculated using the following formula: Cell resistance rate (%) = (experimental group OD_450_ value/control group OD_450_ value) × 100.

#### Western blot test for ABCG2 and Bcl-2 protein expression

Proteins were extracted from the SP and non-SP cells, and protein concentration was determined using the bicinchoninic acid (BCA) method. Following sodium dodecyl sulfate-polyacrylamide gel electrophoresis (SDS-PAGE) and transfer to a membrane, the gels were treated with the primary antibodies (rabbit anti-human ABCG2 and Bcl-2), the secondary antibody (goat anti-rabbit IgG with alkaline phosphatase markers) and a chemiluminescence reagent. The density of the target bands was analyzed using a biomedical image analysis system.

#### Detection of stem cell gene expression by fluorescence quantitative PCR

For RNA extraction, TRIzol was added to samples comprising 2×10^5^ SP and non-SP cells. Amplification was performed by real-time PCR according to the manufacturer’s instructions: 95°C denaturation for 1 min, followed by 95°C for 15 sec, 58°C for 15 sec, and 72°C for 45 sec for a total of 40 cycles. Fluorescence data were collected at the end of each cycle. The 2^−ΔΔCT^ method was used to analyze the Ct value of the target (Musashi-1 and CD44) and reference (β-actin) genes of the two cell types. We calculated the relative gene expression between the SP and non-SP cells by setting the non-SP cell group data as the initial standard. Primers were obtained from Sangon Biotech Co. Ltd. (Shanghai, China), and the sequences used were as follows: forward: 5′-GACTCCAAAACAATTGACCCTAAG-3′ and reverse: 5′-GAGCTTTCTTACATTCCAAACTTT-3′ for Musashi-1; forward: 5′-CGGACACCATGGACAAGTTT-3′ and reverse: 5′-AGCTTTTTCTTCTGCCCACA-3′ for CD44; and forward: 5′-GGACTTCGAGCAAGAGATGG-3′ and reverse: 5′-AGCACTGTGTTGGCGTACAG-3′ for β-actin.

#### In vivo tumor formation

Mice were randomly divided into SP and non-SP groups (n=9 for each group). Each mouse received a subcutaneous injection (in the back) with one of three different cell suspension concentrations: 2×10^2^, 2×10^3^ or 2×10^4^ (n=3 apiece for the SP and non-SP groups). Tumor formation was observed weekly for 8 weeks.

#### Statistical analysis

Data are presented as the mean ± standard deviation. Differences between the two groups were investigated using the Student’s t-test. P<0.05 was considered to indicate a statistically significant difference. All analyses were performed using the Statistical Package for the Social Sciences (SPSS) v.17 (SPSS Inc.; Chicago, IL, USA).

## Results

### 

#### SP cell ratio analysis

**Figure 1:**
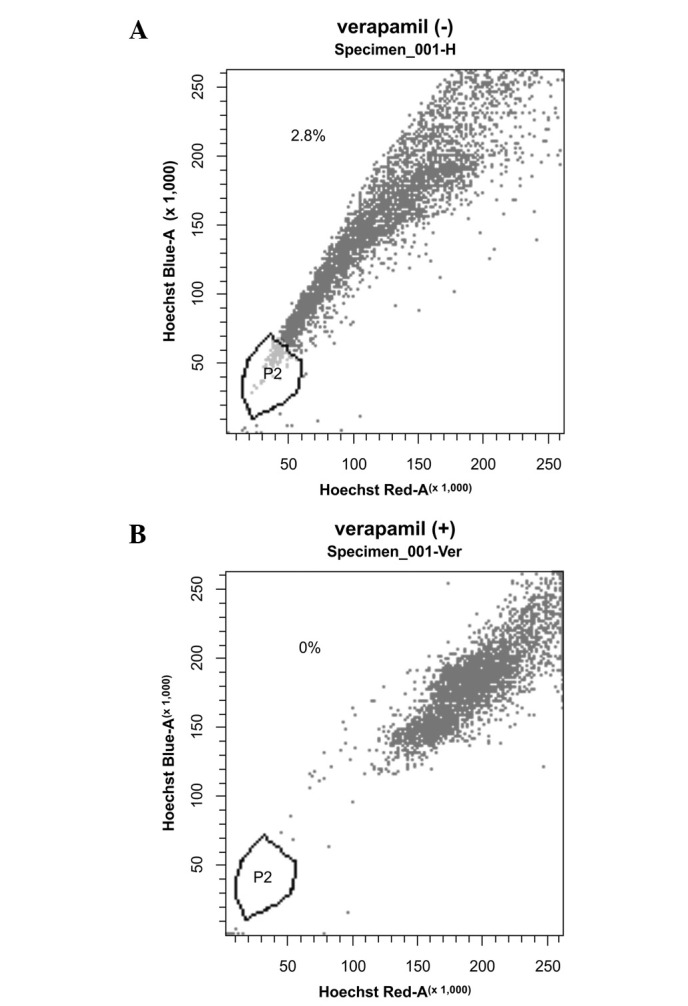
Analysis of side population (SP) cells in the human gastric cancer cell line SGC-7901. Cells were stained using Hoechst 33342 dye and analyzed using flow cytometry. (A) SP cells are outlined and shown as a percentage of the total cell population. (B) The SP cells disappeared in the presence of verapamil.

Only a small proportion (2.3±0.78%) of the SGC-7901 cells were SP cells (Table I). Treatment with verapamil considerably reduced the proportion of SP cells to ∼0% (Fig. 1).

#### In vitro proliferation ability

**Figure 2:**
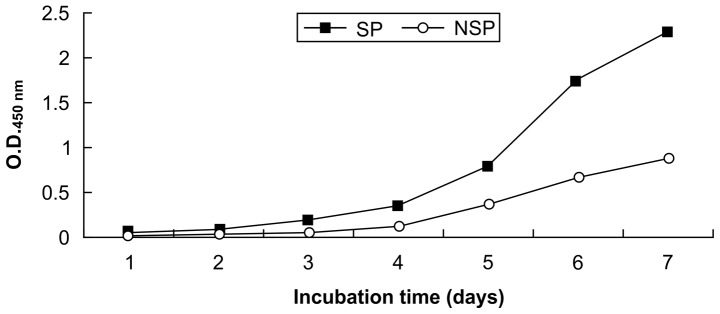
Growth curves for side population (SP) and non-SP (NSP) cells. The x-axis represents time, while the y-axis indicates the corresponding optical density (OD) value at 450 nm.

Fig. 2 shows growth curves for SP and non-SP cells. The SP cells underwent rapid proliferation on day 3, with the growth beginning to plateau by day 7; whereas the non-SP cells exhibited slower growth. The *in vitro* proliferation activity was significantly higher in the SP cells (P<0.05; Table II).

#### Observation of tumor mass formation ability in a serum-free medium

**Figure 3:**
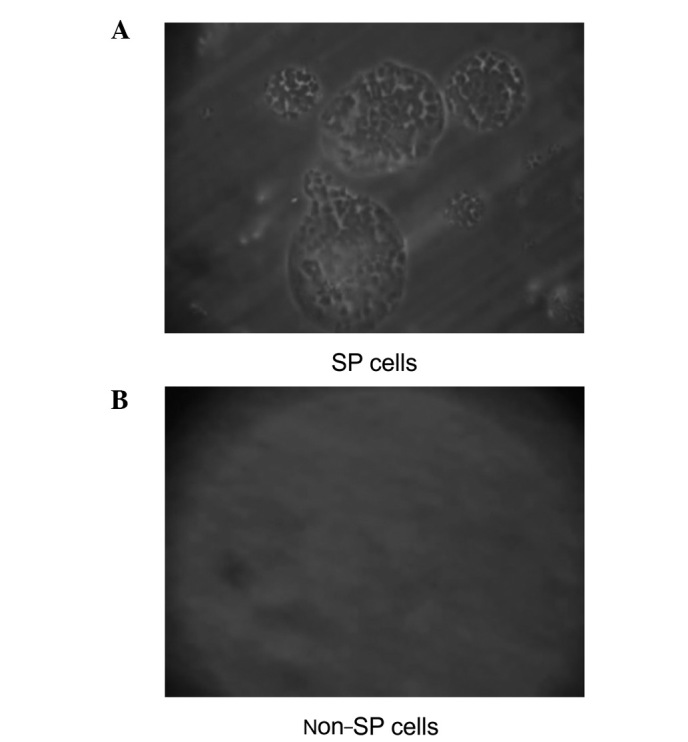
Microscopic observations of serum-free cultures of side population (SP) and non-SP cells. (A) Round and oval ball-shaped suspension tumors are evident in the SP cell group. (B) No such tumors are present in the non-SP cell group.

SP cell cultures contained round and oval ball-shaped suspension tumors characterized by densely packed cells, indicating that these cells have high self-renewal rates in serum-free culture. However, the cells died and no suspension tumors were observed in the non-SP cell cultures (Fig. 3).

#### Appraisal of non-symmetrical division

**Figure 4:**
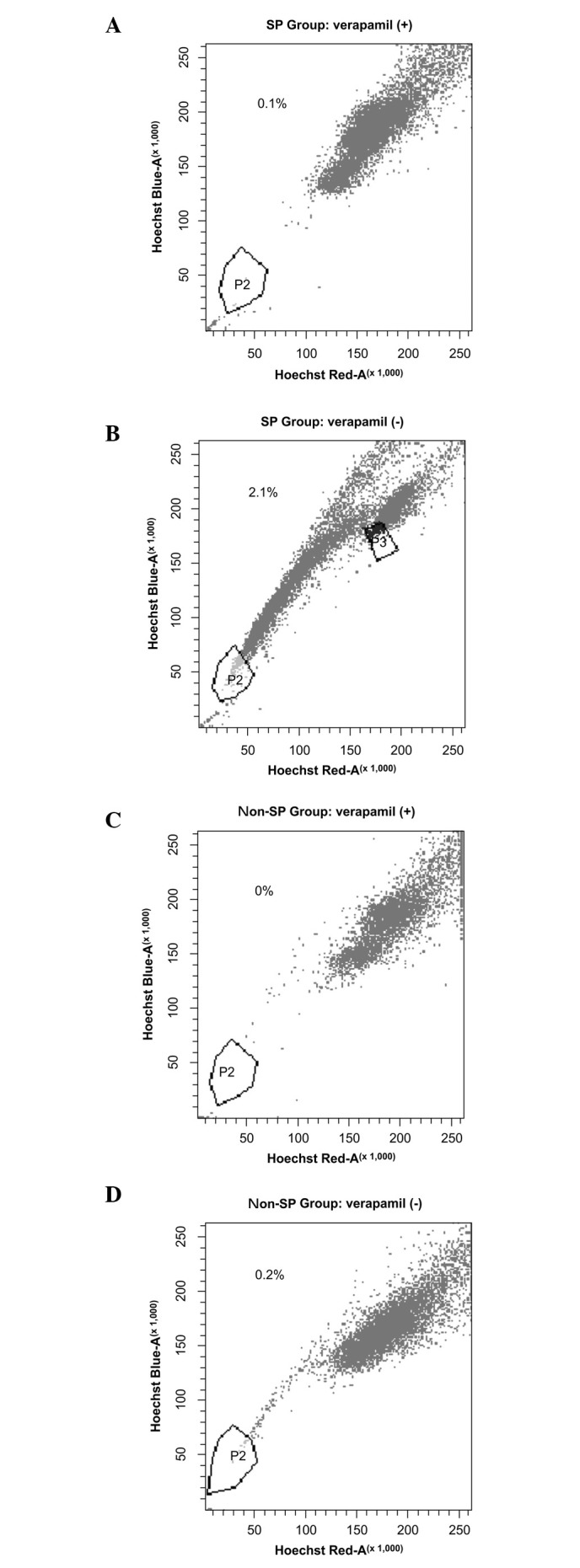
Results of a retest of the two groups of cells. The side population (SP) cells, which disappeared in the presence of verapamil (A), are outlined and (B) shown as a percentage of the total cell population. The non-SP cells, which disappeared in the presence of verapamil (C), are outlined and (D) shown as a percentage of the total cell population.

After one week of cultivation, the ratio of SP cells was 2.1% in the SP group and 0.2% in the non-SP group, suggesting that SP cells exhibit non-symmetrical division. Non-SP cells were also generated during SP cell self-renewal. In the SP cell group, the proportion of SP cells was reduced from 100 to 2.1% (Fig. 4).

#### Cell resistance experiment

**Figure 5:**
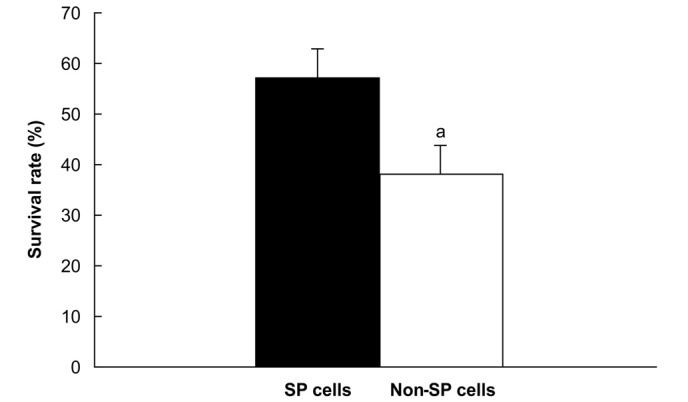
Resistance of side population (SP) cells and non-SP cells to 5-fluorouracil (5-FU). The y-axis shows the survival rate of the two groups following treatment with 10 *μ*g/ml 5-FU for 3 h. ^a^P<0.05

The survival rates following exposure to 10 *μ*g/ml 5-FU were significantly higher for SP cells (57%) than for non-SP cells (38%; P<0.05; Fig. 5).

#### Western blot analysis for ABCG2 and Bcl-2 protein expression

**Figure 6:**
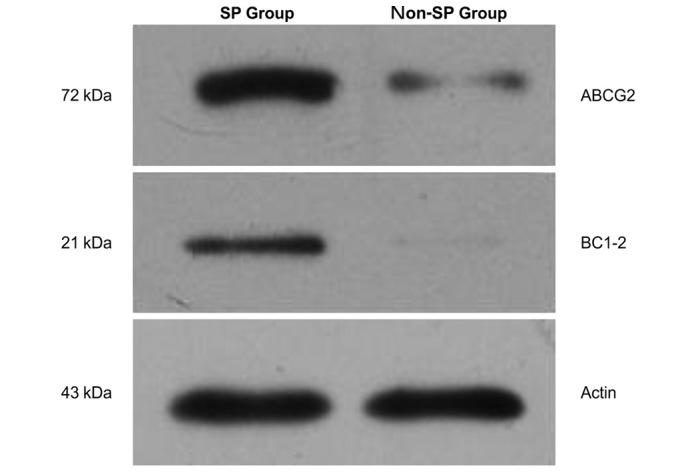
Expression of ABCG2 and Bcl-2 in the two groups. The results show that the ABCG2 strip grey value/reference gray values were 0.99+0.07 and 0.28+0.06, respectively for the SP group and non-SP group; the Bcl-2 strip and reference gray values were 0.47+0.02 and 0.12+0.01, respectively. Therefore, the protein expression levels of ABCG2 and Bcl-2 in SP cells were significantly higher than those in non-SP cells.

The expression levels of ABCG2 and Bcl-2 were higher in SP cells (0.99±0.07 and 0.47±0.02, respectively) than in non-SP cells (0.28±0.06 and 0.12±0.01, respectively; P<0.05; Fig. 6).

#### Detection of stem cell gene expression by fluorescence quantitative PCR

**Figure 7:**
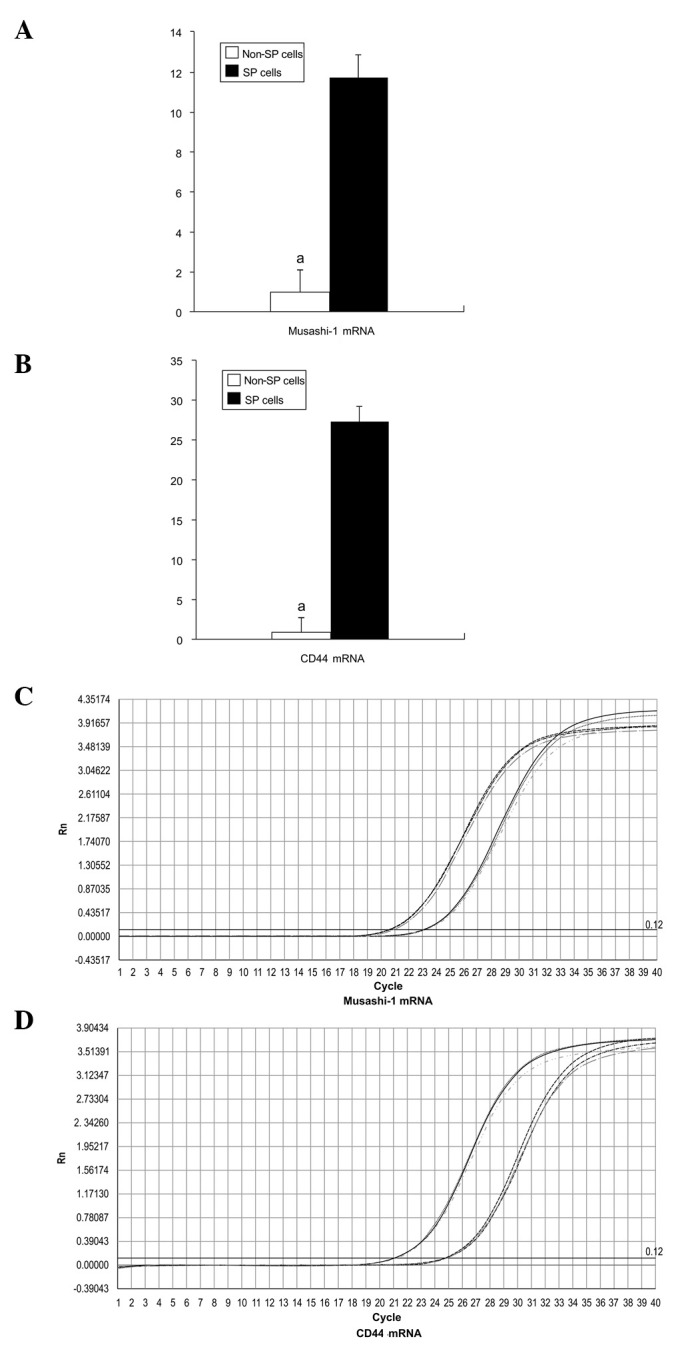
Expression of Musashi-1 and CD44 mRNA in the two groups. The relative expression values of (A) Musashi-1 mRNA and (B) CD44 mRNA (^a^P<0.05). Comparison of amplification curves of (C) Musashi-1 mRNA and (D) CD44 mRNA for the two groups.

The expression of Musashi-1 and CD44 mRNA was significantly higher in SP cells than in non-SP cells, by factors of 11.74 and 27.35, respectively (P<0.05; Fig. 7).

#### In vivo tumor formation

**Figure 8:**
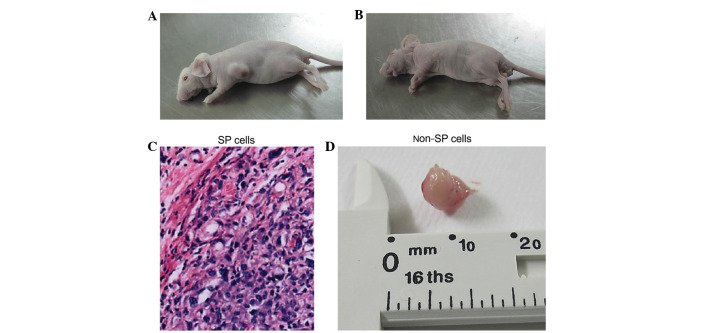
*In vitro* tumor formation in nude mice following the subcutaneous injection of side population (SP) and non-SP cells. (A) Representative subcutaneous tumor produced by the injection of 2×10^4^ SP cells. (B) Lack of subcutaneous tumors resulting from the injection of 2×10^4^ non-SP cells. (C) Representative histopathology image (×100) from an SP tumor. (D) The diameter of the subcutaneous tumor is ∼10 mm.

No tumors were observed in the mice treated with non-SP cell suspensions or in SP-treated mice administered the lowest treatment concentration (2×10^2^ cells). However, one tumor was observed among SP cell-treated mice administered 2×10^3^ cells, and two were observed in mice treated with 2×10^4^ cells (Fig. 8).

## Discussion

We consistently observed differences between SP and non-SP cells, in terms of proliferation and division, survival, gene expression and the ability to form tumor masses. Cumulatively, these results support previous findings (6,7,9,11) that the majority of SP cells in tumors exhibit stem cell characteristics. Notably, the proportion of SP cells found in the current study was slightly higher (2.3±0.78%) than previously reported by Haraguchi *et al* (0.6–2.2%) (6), who identified different proportions of SP cells isolated from four stomach cancer cell strains. These differences may be due to strain-specific variations, the concentration of Hoechst 33343 dye used and/or the FCM settings.

SP cells accounted for just over 2% of all cells in the SGC-7901 cultures. Likewise, after one week of routine culturing, SP cell cultures contained ∼2% SP cells. Proliferation of these cells was observed to be notably more rapid than that of the non-SP cells. Furthermore, SP cells were characterized by the ability to self-renew and form a cell suspension tumor mass, whereas non-SP cells died prior to the formation of tumor masses. Each of these differences may be explained by the peculiar asymmetric splitting abilities exhibited by cancer stem cells; parent cells undergo asymmetric splitting into two daughter cells, one of which retains the biological characteristics of the parent, and the other of which undergoes directional differentiation, thus maintaining its stability and facilitating tumor production.

Cancer therapy is often limited by the resistance of tumors to chemotherapy drugs. SP cells were more resistant to the chemotherapy drug 5-FU and exhibited a significantly higher survival rate than non-SP cells (P<0.05). Similar findings were demonstrated by Fukuda *et al*(11), whose study revealed that SP cells in the gastric cancer cell strain MNK45 exhibited significantly higher resistance to cisplatin chemotherapy drugs and adriamycin than non-SP cells.

Resistance to 5-FU is likely to be facilitated by two major processes, including the activity of adenosine triphosphate binding cassette (ABC) transporters that pump harmful materials out of cells and the suppression of apoptosis. Our results suggest that both processes occur in SP cells. Relative to non-SP cells, SP cells have a significantly higher expression of ABCG2, the most important ABC, and the antiapoptotic protein Bcl-2. Cumulatively, these findings suggest that the chemotherapy resistance characteristics of SP cells are derived from their high expression levels of anti-chemotherapy proteins.

We also observed significantly higher expression levels of Musashi-1 and CD44 mRNA in SP cells compared with non-SP cells. Both products are associated with stem cell behavior. Musashi-1 is an evolutionarily conserved RNA-binding protein that is important in stem cell maintenance, differentiation and the state of tumorigenesis. Nagata *et al*(12) demonstrated that Musashi-1 is found in the stem/progenitor cell area in the mouse stomach. Using a double-marking immunohistochemical method, Akasaka *et al*(13) confirmed the specific expression of Musashi-1 in the distal proliferation area, suggesting that this protein may be the distal stem/progenitor cell surface marker. CD44, on the other hand, was previously speculated to be involved in tumor metastasis involving adhesion molecules between cells. Following its recent identification as a surface marker for cancer stem cells, CD44 has been applied in screening studies concerned with breast (4), prostate (14), pancreatic (15) and head and neck (16) cancer. In a screening study for gastric cancer surface markers, Takaishi *et al*(17) demonstrated that CD44^+^ gastric cancer cells have stronger *in vivo* proliferation and tumor formation ability compared with CD44^−^ cells, suggesting that the former have biological characteristics similar to those of tumor stem cells. The high expression of both of these types of mRNA in SP cells suggests that gastric cancer stem cell subsets may exist in SP cells.

We used xenotransplantation techniques to confirm the patterns suggested by our observational work, particularly that SP cells are correlated with higher rates of tumor formation. Subcutaneous injection of non-SP cells failed to produce any tumors within 8 weeks, while both the 2×10^3^ and 2×10^4^ SP doses were associated with tumor formation (one and two tumors, respectively).

Overall, our findings indicate that a small number of SP cells exist in the gastric cancer strain SGC-7901. Relative to non-SP cells, these cells have a stronger *in vitro* proliferation ability, a stronger renewal ability, non-symmetrical division, greater resistance to chemotherapy, higher expression of stem cell genes, and the capacity for *in vivo* and *in vitro* tumor formation. These results further demonstrate that SP cells isolated from the gastric cancer cell strain SGC-7901 exhibit biological characteristics similar to those of cancer stem cells. Thus, we conclude that SP cells are rich in gastric cancer stem cell subsets, and that the SP cell sorting method is an effective means for isolating and identifying gastric cancer stem cells in early screening. It is difficult to identify gastric cancer stem cells, as they are few in number and their specific surface markers remain unknown. Therefore, further DNA microarray analyses using SP cells are required to identify potential candidates for gastric cancer stem cells markers, which may contribute to a more accurate targeted therapy for gastric cancer.

## Figures and Tables

**Table I:** Results from the SP cell detection test.

Sample number	Percentage of SP cells
1	1.9
2	2.8
3	3.2
4	2.4
5	1.2

SP, side population.

**Table II:** OD_450_ values of the two groups at different incubation times.

	Time (days)						
Group	1	2	3	4	5	6	7
SP cells	0.0451±0.0073	0.0837±0.0052	0.1872±0.0045	0.3527±0.0062	0.7834±0.0193	1.7483±0.0782	2.2657±0.09531
Non-SP cells	0.0274±0.0059	0.0468±0.0027^a^	0.0732±0.0061^a^	0.1368±0.0057^a^	0.3829±0.0094^a^	0.6746±0.0629^a^	0.8837±0.06382^a^

Values are presented as the mean ± standard deviation;

a P<0.05. OD_450_, optical density at 450 nm; SP side population.
